# Appendiceal diverticulum diagnosed after appendectomy: Two case reports and literature review

**DOI:** 10.1016/j.ijscr.2025.110916

**Published:** 2025-01-20

**Authors:** Souad Ghattas, Faten Mohtar, Jad El Bitar, Georges Gandour, Marwan Haddad, Nazem Matta

**Affiliations:** a Department of General Surgery, Mount Lebanon Hospital University Medical Center, University of Balamand, Beirut, Lebanon; b Head of Radiology Department, Mount Lebanon Hospital University Medical Center, University of Balamand, Beirut, Lebanon

**Keywords:** Appendiceal diverticulitis, Acute appendicitis, Appendectomy, Diverticulum, Abdominal pain, Case report

## Abstract

**Introduction:**

Appendiceal diverticulitis is an uncommon pathology that imitates acute appendicitis and is usually treated by appendicectomy.

**Cases presentation:**

We present two cases: a 50-year-old female patient and a 35-year-old male patient, both of whom presented with signs and symptoms of acute appendicitis and were managed accordingly. Final pathological examination confirmed the presence of an appendiceal diverticulum.

**Clinical discussion:**

Diverticulitis of the appendix is four times more likely to lead to perforation when compared to appendicitis and may be associated with underlying neoplasm. Therefore, it is extremely important to distinguish diverticulitis of the appendix from appendicitis.

**Conclusion:**

Clinicians should always consider appendiceal diverticulitis when evaluating patients with right-sided, lower quadrant abdominal pain.

## Highlights

• Diverticulosis of the appendix is a very rare pathology with an incidence reported to be between 0.004% and 2.1%.
• Discussion: Diverticulitis of the appendix is four times more likely to lead to perforation when compared to appendicitis and may be a sign of underlying neoplasm.
• Clinicians should always keep appendiceal diverticulitis in mind when treating patients with right-sided, lower quadrant abdominal pain.

## 1 Introduction

Appendiceal diverticulosis is a very rare pathology with an incidence reported to be between 0.004 % and 2.1 % [1]. They are false diverticula caused by mucosal herniation through the deficient muscularis propria [2]. Diverticular disease of the appendix often presents clinically similar to acute appendicitis and its usually treated with appendectomy. Therefore, appendiceal diverticulitis is generally diagnosed after the pathological examination [2]. The work has been reported in line with the SCARE criteria [3].

We present here two cases of patients who were initially treated for acute appendicitis, only to have appendiceal diverticula identified on subsequent pathology reports.

## 2 Case 1

We present the case of a 50-year-old female patient with a history of iron deficiency anemia, who presented with diffuse abdominal pain of 1-week duration. The pain was colicky in type and had increased in intensity over the past two days, becoming more localized to the right lower quadrant. This pain was not related to food, and the patient had not experienced similar episodes before. She denied nausea, vomiting, diarrhea, or constipation, and also had no fever, chills, or urinary or respiratory symptoms. Her past surgical history included a Cesarean section. She has no known allergies, and smokes one pack of cigarettes per day. Upon arrival at the emergency room, her vitals were within normal limits and she was afebrile. On physical examination, her abdomen was soft, non-distended with diffuse tenderness and severe right lower quadrant tenderness with positive McBurney's sign.

Her labs were as follows: Normal WBC: 5.35 × 10^3/ul, RBC: 5.11 × 10^6/ul, Hb: 14.2 g/dl, Hct: 43.6 %, platelets: 241 × 10^3/ul, slightly elevated CRP: 16 mg/dl. Urinalysis results were negative.

**Fig. 1:**
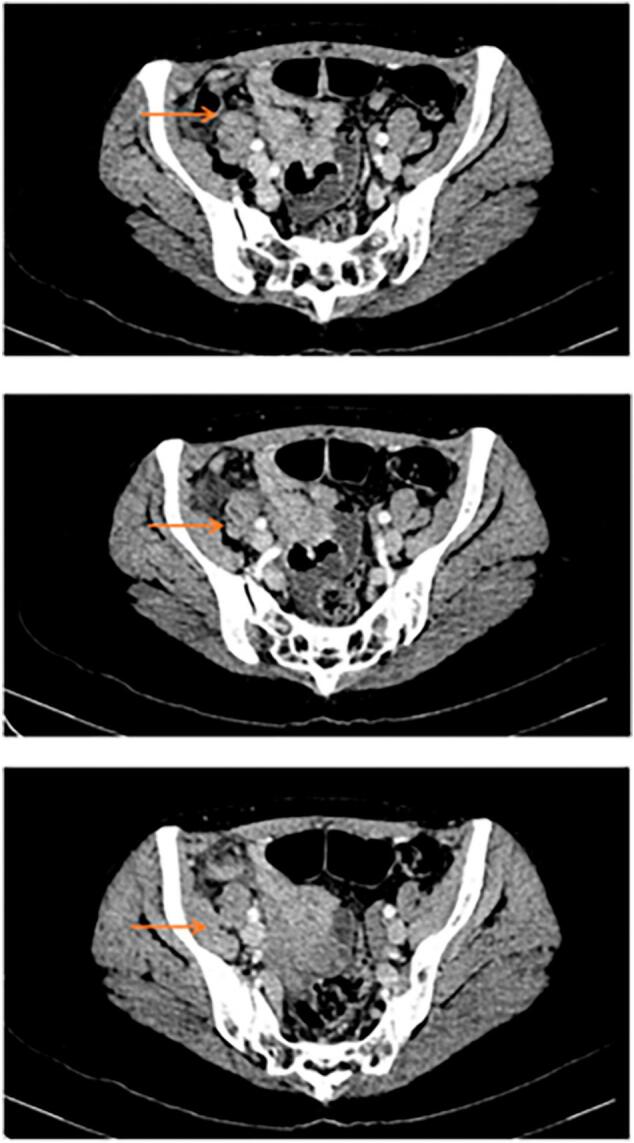
Ct abdomen and pelvis with IV contrast, Transversal View, showing inflamed appendix (orange arrows). (For interpretation of the references to colour in this figure legend, the reader is referred to the web version of this article.)

CT scan of abdomen and pelvis with IV contrast (Fig. 1) was done that showed a dilated appendix with edematous and enhanced wall, measuring up to 13 mm surrounded by fat streaking. There was mild free fluid, but no evidence of abscess. The rest of the scan was unremarkable. These findings were suggestive of acute non-complicated appendicitis, and the patient was admitted to the hospital. She was started on Ceftriaxone and Metronidazole, and underwent laparoscopic appendectomy the next day. The surgery was straightforward, with no complications intraoperative, and the specimen was sent to pathological examination. Postoperatively, the patient had an uneventful recovery, was discharged on postoperative day one with pain medications, and was prescribed oral Cefuroxime for one week.

Two weeks later, the pathology report was received. The gross description described an appendix measuring 9.5 × 2.5 cm with a 1 × 0.7 cm diverticulum at the tip. The microscopic description, the diverticulum is lined by a focally ulcerated mucosa. The wall is expanded by a purulent leucocytic exudate, and the outer surface is covered by a fibrino-leucocytic material.

## 3 Case 2

We present a case of a 35-year-old male patient with a history of migraine headache, who presented with right lower quadrant pain that started a week ago. He described the pain as colicky not related to food. The pain increased two days ago and started radiating to the back. He denied any nausea, vomiting, diarrhea, or constipation. He also didn't have any fever, chills, urinary or respiratory symptoms. Past surgical history included surgery for gynecomastia. He takes Paroxetine as prophylaxis for his migraine. He is a non-smoker, non-alcoholic and has no food or drug allergies. On physical examination, abdomen was soft, non-distended with localized right lower quadrant tenderness and positive McBurney's sign.

His labs were as follows: WBC: 7.98 × 10^3/ul, RBC: 4.79 × 10^6/ul, Hb: 14.7 g/dl, Hct: 40.3 %, platelets: 153 × 10^3/ul, C-reactive protein (CRP): 19 mg/dl (slightly elevated), SGPT: 16 IU/l, GGT: 13 IU/l, lipase: 8 IU/l. Urine analysis came back negative.

**Fig. 2:**
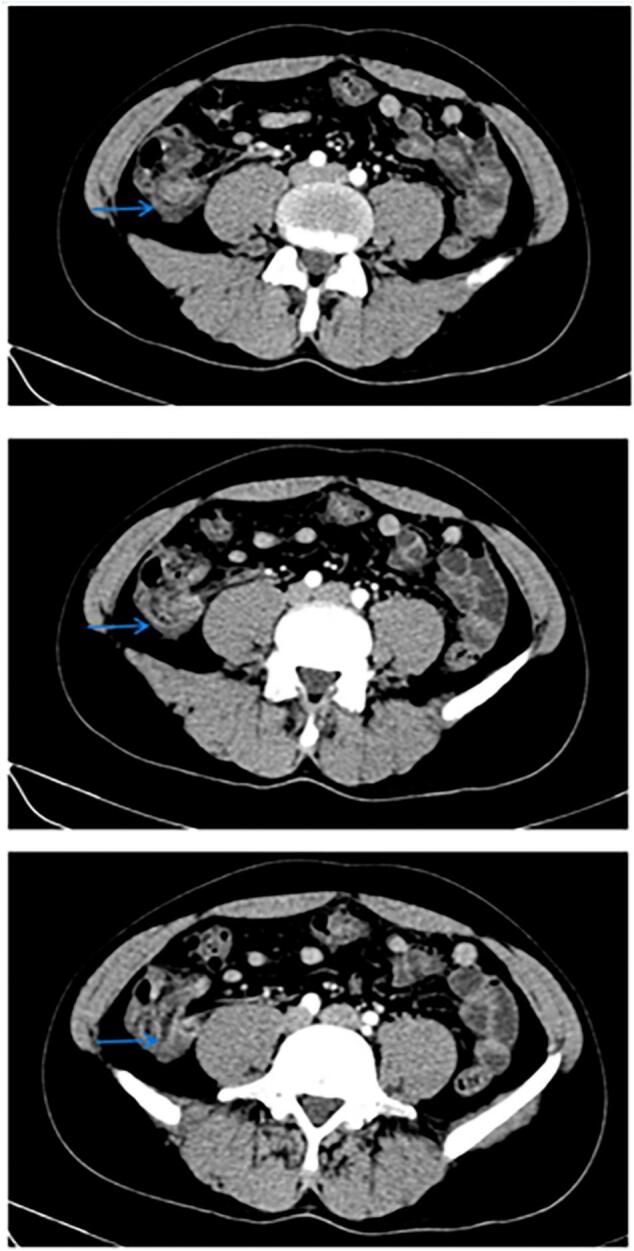
Ct scan abdomen and pelvis with IV contrast, Transversal View, showing inflamed appendix (blue arrows). (For interpretation of the references to colour in this figure legend, the reader is referred to the web version of this article.)

CT scan of abdomen and pelvis with IV contrast (Fig. 2) showed a dilated appendix up to 14 mm, fluid-filled with thickened enhancing and irregular walls surrounded by fat stranding and mild amount of fluid. These findings were suggestive of acute pre-perforative appendicitis. The patient was admitted to the hospital and started on IV antibiotics: ciprofloxacin and metronidazole. Uncomplicated laparoscopic appendectomy was done the next day and the patient was discharged the day after.

The gross pathological description of the specimen showed an appendix measuring 9 × 1.5 cm with the tip showing a diverticulum surrounded by a thick wall. Microscopically, there is a diverticular pouch lined by an unremarkable colonic mucosa. The latter is focally ulcerated and the wall is expanded by a polymorphic leucocytic exudate. The surrounding subserosa is thick, fibro-edematous and expanded by a polymorphic leucocytic population (Table 1).

**Table 1:** Summarizing the two cases described.

Case	Case 1	Case 2
Age	50	35
Gender	Female	Male
Symptoms	Diffuse colicky abdominal pain that became localized to right lower quadrant	Right lower quadrant pain
Imaging findings	CT scan abdomen and pelvis with IV contrast showed dilated appendix with edematous and enhanced wall, measuring 13 mm with fat stranding, no abscess.	CT scan abdomen and pelvis with IV contrast showed a dilated, fluid-filled appendix measuring 14 mm with wall thickening and fat stranding reaching the ileocecal junction and terminal ileum with mild reactive peritonitis
Pathology results	Appendix measuring 9.5 × 2.5 cm with a 1 × 0.7 cm diverticulum at the tip	Appendix measuring 9 × 1.5 cm with the tip showing diverticula surrounded by a thick wall

## 4 Discussion

Appendiceal diverticulosis can be classified into congenital and acquired types [4]. The congenital type occurs due to the outpouching of all three appendiceal layers through a normal wall. This usually occurs on the antimesenteric border of the appendix and may be associated with disease such as Patau syndrome [4].

The acquired type diverticula occur secondary to increased pressure within the appendiceal lumen due to the presence of a fecalith, proximal tumors or excess mucus, leading to an outpouching of the mucosa through the muscularis propria at low-pressure points along the mesenteric and antimesenteric borders. Acquired diverticula are often multiple, located in the distal 1/3 of the appendix and they are classified as false diverticula [4]. Our patients were found to have a diverticulum at the tip of the appendix on the post operative pathology.

Risk factors associated with diverticular disease of the appendix include male gender, ager over than 30 years, cystic fibrosis, and Hirschsprung disease [2]. The risks factors present in our patients were age for the two patients 50 and 35 years old, and male gender for the second patient.

In 1989, Lipton et al. classified the morphological types of appendiceal diverticular disease into four categories: type I includes the presence of acute diverticulitis with a normal appendix, type II describes acute diverticulitis with acute appendicitis, type III is a non-inflamed diverticulum with appendicitis, and type IV is a non-inflamed diverticulum with a normal appendix. Types I-III are also sub-grouped into those with or without perforation [5]. Our patients were classified as type III diagnosed on pathology results.

Symptoms of diverticulitis of the appendix are similar to those of acute appendicitis but diverticulitis of the appendix is four times more likely to lead to perforation when compared to appendicitis and may be a sign of underlying neoplasm [6]. Therefore, it is extremely important to distinguish diverticulitis of the appendix from appendicitis. Our patients presented with signs and symptoms of acute appendicitis and were managed accordingly.

The perforation rate in appendiceal diverticulitis ranges from 30 % to 70 %, which is more than four times higher than that of appendicitis. This increased risk is attributed to the thin-walled nature of the diverticulum, making it more prone to rupture [6].

Several studies have found a significant association between appendiceal diverticula and epithelial neoplasms, highlighting the importance of post-appendectomy pathology for patients with incidental appendiceal diverticula. Appendiceal diverticula is associated with underlying neoplasms in 7.1 % to 48 % of cases including carcinoids, mucinous adenomas, tubular adenomas and adenocarcinomas [7].

CT is the imaging modality of choice for identifying appendiceal diverticular disease. However, preoperative radiological diagnosis is often missed in practice [8]. A contributing factor is likely und er-recognition of the condition and its significance. The key imaging differentiation is the identification of a diverticula arising from the appendix at the epicenter of the inflammatory change, peri-appendiceal fat stranding, a larger appendiceal diameter, the saccular structure of the appendix wall, cecum or ascending colon diverticulum, and peri- appendiceal or peri-cecal fluid collection [7]. In our cases the identification of the diverticula was difficult due to the advanced inflammatory changes of the appendix.

Regarding management, there is increasing evidence supporting early appendectomy for both appendiceal diverticulitis and diverticulosis. Elective appendectomy is recommended for incidentally detected diverticular disease of the appendix due to the high risk of diverticulitis, perforation, and development of appendiceal malignancy risk [6].

Appendiceal diverticula are commonly identified incidentally post appendectomy for patients with a clinical presentation of acute appendicitis [9,10].

The incidence of Appendiceal diverticula could be higher than given in the literature [1]. More careful pathological processing of the specimens is needed. Moreover, the duration of the disease is longer for Appendiceal diverticula than acute appendicitis [5,6,11].

## 5 Strengths and limitations

The article provides an insightful exploration of appendiceal diverticulitis, with clear case studies, practical clinical recommendations, and a strong discussion of the pathology, risk factors, and management. Its focus on a rare condition and the importance of proper diagnosis makes it valuable for clinicians. However, the article could be strengthened by acknowledging the limitations of the presented cases. For example, the cases are relatively small, and more evidence from a broader cohort would help confirm the findings. Additionally, addressing potential biases (such as selection bias) and the limitations of preoperative imaging in detecting appendiceal diverticulitis could provide a more balanced perspective.

## 6 Implications for future research

It would be beneficial to enrich the medical field by considering further research to refine the management guidelines for appendiceal diverticulitis and to explore the long-term outcomes of prophylactic appendectomy.

## 7 Conclusion

Appendiceal diverticulitis is a rare condition that presents similarly to acute appendicitis and is typically treated with appendectomy. Prophylactic appendicectomy should be considered for non-inflamed diverticula. This is due to the risk of possible inflammation in the future, risk of perforation, and risk of developing an appendiceal neoplasm. Clinicians should consider appendiceal diverticulitis in mind when treating patients with right-sided, lower quadrant abdominal pain.

## Author contribution

**Souad Ghattas** (first Author, conception of the work, design of the work, revising the work critically for important intellectual content, final approval of the version to be published), **Faten Mohtar** (review of literature, conception of the work, design of the work, revising the work critically for important intellectual content, final approval of the version to be published), **Jad El Bitar** (conception of the work, design of the work, review of literature, draft manuscript,), **Georges Ghandour** (review of literature, draft manuscript), **Marwan Haddad** (supervision, validation, project administration, conception of the work, revising the work critically for important intellectual content, final approval of the version to be published project administration ), **Nazem Matta** (supervision, validation, project administration, conception of the work, revising the work critically for important intellectual content, final approval of the version to be published project administration and corresponding author).

## Consent

Written informed consent was obtained from the patient for publication and any accompanying images. A copy of the written consent is available for review by the Editor-in-Chief of this journal on request.

## Ethical approval

Case report approved for publishing by ethical committee at Mount Lebanon Hospital, University Medical Center, Lebanon, Beyrouth on 1 Octobre 2024.

## Guarantor

Souad Ghattas.

## Research registration number

Not applicable.

## Provenance and peer review

Not commissioned, externally peer-reviewed. This work has been reported in line with the SCARE criteria: Sohrabi C, Mathew G, Maria N, Kerwan A, Franchi T, Agha RA. The SCARE 2023 guideline: updating consensus Surgical Case Report (SCARE) guidelines. Int J Surg Lond Engl. 2023;109(5):1136.

## Funding

None.

## Conflict of interest statement

The authors report no conflict of interest.
